# Plug-and-play round-robin differential phase-shift quantum key distribution

**DOI:** 10.1038/s41598-017-15777-9

**Published:** 2017-11-13

**Authors:** Qian-Ping Mao, Le Wang, Sheng-Mei Zhao

**Affiliations:** 1 Institute of Signal Processing and Transmission, Nanjing University of Posts and Telecommunications, Nanjing, 210003 China; 2 College of Computer Science and Technology, Nanjing Tech University, Nanjing, 211800 China

## Abstract

The round-robin differential-phase-shift quantum key distribution (RRDPS-QKD) protocol could provide an effective way to estimate the leakage information without monitoring the signal disturbance. Moreover, the self-compensating property of plug-and-play (P&P) setup can eliminate the variations of phase or polarization in QKD procedure. In the paper, we introduce the P&P concept into RRDPS-QKD, and propose a QKD protocol, named P&P RRDPS-QKD protocol, to make the RRDPS-QKD scheme more practical. We analyze the security, and discuss the key generation rate with infinite-intensity decoy state method. The results show that the proposed protocol is a good solution to RRDPS-QKD protocol with untrusted sources. It has a high security and its key generation rate could be as good as the protocol with trusted sources when the average input photon number *N* is greater than 10^6^. In addition, the proposed protocol has a high noise tolerance in comparison with P&P BB84-QKD protocol.

## Introduction

Quantum key distribution (QKD) allows two distant parties (Alice and Bob) to share secret keys even with the existence of an eavesdropper, Eve^1^. The unconditional security is based on quantum mechanics, such as quantum no-cloning theorem and Heisenberg’s uncertainty principle^2–4^. Since the first QKD (BB84-QKD) was proposed^1^, many QKD protocols have been presented to enhance the security of the practical quantum communications, such as, decoy-state QKD protocol^5–7^, device-independent QKD protocol^8^ and measurement-device-independent QKD protocol^9–14^. The security proofs of the above QKD protocols are focused on the amount of the information leaked to Eve. According to the Heisenberg’s uncertainty principle, any intervention from Eve would inevitably cause the disturbance in the quantum signals, and the leakage information can be estimated by monitoring the signal disturbance^15–17^. Recently, Sasaki *et al*. proposed a new QKD protocol, named round-robin differential-phase-shift QKD (RRDPS-QKD)^18^, where the information leakage is estimated without any monitoring, but depends only on the state prepared by Alice.

In RRDPS-QKD protocol, Alice encodes random bits in the phases of quantum signal including *L* pulses, named train, and sends the signal to Bob through an insecure quantum channel. Then Bob randomly picks two pulses in a train, and measures the relative phase between them to obtain the raw key. Bob’s randomness makes it hard for Eve to obtain the information of the key, while the leakage information only comes from the state prepared by Alice. With large enough *L*, the tolerance of bit error rate *e*_*bit*_ can be up to 50%. It becomes a promising practical QKD scheme.

Up to now, both theoretical^19–26^, and experimental^27–30^ studies of RRDPS-QKD have been developed. Zhang *et al*.^25^ applied the tagging technique to overcome the effects of background noise and misalignment. Wang *et al*.^29^ presented an active implementation of the protocol, where secret keys can be distributed over the distance of 90 km. Constructing a variable-delay interferometer with 127 actively selectable delays, Li *et al*.^30^ experimentally demonstrated the RRDPS protocol and obtained a final key rate of 15.54 bps with a total loss of 18 dB and an error rate of 8.9%.

To guarantee the security of RRDPS-QKD protocol, the sources are usually assumed to be the trusted ones. However, this assumption is not always valid in practice. For example, the intensity fluctuation from the laser source, the parameter fluctuation from the optical devices, and the birefringence of the fibers may invalidate the assumption^31^. Hence, it is still a challenge to assure the security of RRDPS-QKD protocol with an untrusted source.

On the other hand, a plug-and-play (P&P) QKD setup is often used to QKD to avoid the careful adjustments and the control of the system on both sides of the communication channel^32^, and the self-compensating property could eliminate the variations of phase or polarization in the QKD procedure^32,33^. Furthermore, the QKD protocol with P&P configuration can give the key generation rate closely to that with a trusted source, even if the source is unknown and untrusted, which is equivalent to the source controlled by an eavesdropper^34–37^.

In this paper, we propose a P&P RRDPS-QKD protocol to make RRDPS-QKD be more practical. In the protocol, the trains of *L* strong optical pulses, instead of weak ones, are prepared by Bob, and then are encoded and attenuated to single-photon levels by Alice. Later, the weak pulses carrying information are sent back to Bob with a Faraday mirror and measured by Bob’s variable-delay interferometer. Lastly, Alice and Bob could obtain a secret key with the indices of successful measurement results, together with error correction and privacy amplification. In addition, the security is analysed and the lower bound of the key generation rate is estimated. The performance comparison between our protocol, one-way RRDPS-QKD with a trusted source and the P&P BB84-QKD with an untrusted source are also presented.

The proposed protocol has the following advantages: (i) The assumption of a trust source can be removed. (ii) Due to the self-compensating property of the bidirectional structure, the protocol has a high stability. (iii) Strong optical pulses are utilized. This ensures that the preparation, monitoring and synchronization of the source are easily realized under the proposed technique. (iv) The P&P architecture doesn’t disturb the measurement setup of the RRDPS protocol, so the high tolerance of bit error rate in RRDPS protocol is maintained. (v) The proposed protocol is more practical.

## Results

### The scheme of the plug-and-play RRDPS-QKD

**Figure 1:**
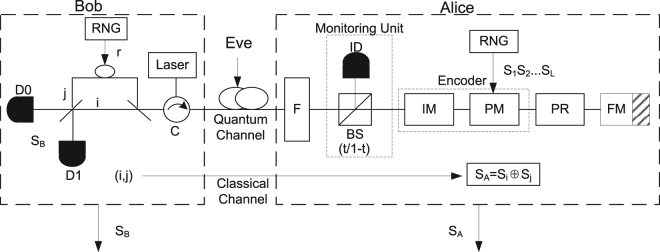
The schematic diagram of the P&P RRDPS-QKD protocol. D0 and D1 are two single-photon detectors. RNG is a random number generator. C is a circulator. Laser represents strong pulses source. F is an optical filter. A monitoring unit consists of a beam splitter (BS) and a classical intensity detector (ID). Intensity modulator (IM) and phase modulator (PM) constitute an encoder. PR is a phase randomizer. FM is a Faraday mirror.

The schematic diagram of the proposed P&P RRDPS-QKD is shown in Fig. 1. A series of *L*-pulse trains are generated by a strong laser from Bob and transmitted through a quantum channel to Alice. Once the trains arrive at Alice, they are passed through an optical filter (F) and a monitoring unit, which consists of a beam splitter(BS) and an intensity detector (ID). After being reflected by a Faraday mirror (FM), they are phase randomized by a phase randomizer (PR), and encoded by an encoder that consists of an intensity modulator (IM) and a phase modulator (PM). The IM attenuates the optical pulses and sets the average photon numbers to the desired signal or decoy states. Here, the devices (PM, PR and IM) are properly designed to work only during a short time that the legitimate signal is passed. The weak pulses carrying information are sent back to Bob. Bob’s measurement is an unbalanced Mach-Zehnder interferometer (MZI) with a variable delay *r* controlled by a random number generator (RNG). Using the MZI, Bob detects the signal and acquires the indices {*i*, *j*}, and announces the indices via a public channel to Alice. Then Alice and Bob obtain a sifted key *s*_*A*_ and *s*_*B*_, respectively. Finally, after performing error correction and privacy amplification, Alice and Bob can share a secure key.

#### The proposed protocol

The details of our protocol are as follows.

1. Pulse trains preparation and transmission. Adopting a strong laser source, Bob (the receiver of signal), instead of Alice (the sender of signal), prepares a series of pulse trains each containing *L* optical pulses and sends them to Alice through an optical fiber.
2. Trains monitoring and information encoding. Once each train arrives, Alice performs the filtering by the optical filter (F), and monitors the train by the monitoring unit. Then the pulse trains are passed and reflected from a Faraday mirror (FM) and randomized by a phase randomizer (PR). Alice encodes her information on the series of random *L*-bit sequences is already generated by Alice with the RNG in her side, and each symbol of one sequence ${s}_{1}{s}_{2}\ldots {s}_{L}$ is encoded to different phases modulated on the *L* pulses in one train with the phase modulator (PM), for instance, *s*_*i*_ = 0 with 0 phase, *s*_*i*_ = 1 with *π* phase. Using the IM, Alice attenuates heavily the pulse train to quantum level, and randomly generate signal state with intensity *μ* or decoy states with intensities ${\nu }_{1}{\nu }_{2}\ldots {\nu }_{m}$. Finally she sends the encoded trains back to Bob.
3. Measurement. Upon receiving the *L*-pulse train, Bob splits the *L*-pulse train into two *L*-pulse trains with a 50:50 beam splitter (BS). Then he uses the other RNG to generate a random number $r\in \{-L+1,\cdots ,-2,-1,1,2,\cdots ,L-1\}$, and shifts one of the *L*-pulse train by *r* pulses. Bob Measures the interference between the unshifted *L*-pulse train and the r-delayed train. If Bob obtains a detection result on position *i* in the unshifted *L*-pulse train, corresponding to position *j* in the shifted *L*-pulse train, where *i* and *j* satisfy ${j}={i}+r{(}\mathrm{modL}{)}$, Bob records the result as *s*_*B*_ according to the responses of Bob’s detectors (*s*_*B*_ = 0 according to a click in D0 or *s*_*B*_ = 1 according to a click in D1). Otherwise, Bob regards the transmission as a failure and discards it.
4. Key sifting. Bob announces the indices {*i*, *j*} to Alice through a classical channel, and Alice obtains a sifted key bit *s*_*A*_ by computing ${s}_{A}={s}_{i}\oplus {s}_{j}$.
5. Error correction and privacy amplification. After repeating steps (1)–(4) to accumulate enough sifted key bits, Alice and Bob perform error correction and privacy amplification on the sifted key to extract the final secure key.

#### Security

Next, we discuss the security of the proposed protocol from two aspects. One aspect is the security of the protocol itself.

In the above procedures, three skills are employed by Alice to enhance the security. First, the narrow bandpass filter is adopted in the proposed protocol to allow only a single mode to enter the encoder, therefore, the single-mode assumption for each signal is guaranteed^37,34^. Secondly, the monitoring unit is employed to detect the pulse energy and the arrival time to acquire certain information about the photon-number distribution and the timing mode. By randomly sampling a portion of the pulses to test the photon numbers, the bounds on the output photon-number distribution can be estimated^34,37,38^. Finally, the phase randomizer is used to make the phases of the *L*-pulse trains completely random, which can guarantee the phase randomization assumption made in the security proof for laser-based QKD^5,39,40^.

On the other hand, as Bob’s random measurement is after Eve’s disturbance, the information leaked to Eve is very limited because of information causality^41^. Intuitively, Eve seems to have some control over the generation of index *i*, but the other index *j* is determined randomly from the rest of the *L* − 1 as $j=i+r(modL)$ by the random number *r*. According to the security proof of the original RRDPS^18^, the possibility of Eve’s successful interference in the particular value ${s}_{i}\oplus {s}_{j}$ is in the order *O*(*L* ^−1^), which means that the leakage information is dependent on the number of pulses of the source, *L*. Hence, the amount of privacy amplification depends only on the source and there is no need to monitor Eve’s disturbance.

The other aspect is the security against typical attacks. For the QKD protocol, there are several eavesdropping strategies for Eve to obtain key information. In the following, we analyze them, individually.

(i) The beam splitting attack: During the protocol, Eve has two chances to intervene and eavesdrop the signal by beam splitting attack. The first chance is the transmission of strong pulse trains from Bob to Alice. Since there is no information encoded on the beam, Eve can not eavesdrop any information at this stage. The other chance is the transmission of the encoded weak pulse trains back from Alice to Bob. Because these *L*-pulse trains have been attenuated to the single-photon level, Eve has no control over which of the pulses the photons are in and has a very small possibility to tap one photon if a pulse happens to have more than one photon. Hence she cannot aim to learn the phase difference between a specific pair of pulses. Neither can she force Bob to announce a particular values of {*i*, *j*}, since the difference *r* is randomly chosen by Bob. Such a two fold randomness in {*i*, *j*} makes the eavesdropping difficult. So the present protocol is safe under the beam splitting attack.
(ii) The Trojan-horse attack^38^. Ideally, Eve may send a spying pulse to Alice to detect the phase shift. However, the pulses from Bob are greatly attenuated by Alice to the single-photon level, typically, 0.1 per pulses. To get the spying pulse returned with at least 1 photon per pulse, Eve has to send her pulse ten times stronger than Bob’s, which means that Alice can easily detect the existence of Eve by monitoring the pulse energy. So the monitoring unit is also useful in preventing a Trojan-horse attack.
(iii) The IR(intercept/resent) attack: For this type of attack, Eve may intercept/resent the signal by using the same receiver’s setup as Bob, and detects a photon as Bob does. However, she only could obtain the partial information of index *i*, because Bob has not yet announced the indices {*i*, *j*}. In order to find the secret key, Eve has to resend a fake pulse train to Bob, which unfortunately for Eve her existences will be revealed by an obvious increase of error rate.

#### Key rate

Define N and K as the average photon number of the source to Alice and the number of pulses, respectively. Here, N and K are the larger positive integers. In the QKD protocol with an untrusted source^34,36,37^, the pulses with photon number $m\in [(1-\delta )N,(1+\delta )N]$ are defined as “untagged” bits, and the other pulses with photon number $m < (1-\delta )N$ or $m > (1+\delta )N$ are defined as “tagged” bits, where *δ* is a small positive real number chosen by Alice and Bob. According to the random sampling theorem, if Δ is defined as the average probability of the tagged sampling pulses in the asymptotic case, Alice can conclude that there are no fewer than $(1-{\rm{\Delta }}-{\varepsilon })K$ untagged encoding pulses with high fidelity, where *ε* should be satisfy that ${\varepsilon }^{2}K\gg 1$. Therefore, Alice and Bob focus only on the $(1-{\rm{\Delta }}-\varepsilon )K$ untagged bits for key generation of the protocol. The key rate of the protocol should be

1 $$R=\frac{1}{L}\{(1-{\rm{\Delta }}-\varepsilon )Q-{Q}_{e}fH({e}_{bit})-(1-{\rm{\Delta }}-\varepsilon )Q{H}_{PA}\}.$$

where *Q*_*e*_ and *e*_*bit*_ denote the overall gain and the quantum bit error rate (QBER), respectively. *f* denotes the efficiency of the error correction. $H(x)=-{\rm{x}}{\mathrm{log}}_{{\rm{2}}}(x)-(1-x){\mathrm{log}}_{{\rm{2}}}(1-x)$ is the binary Shannon entropy function. *H*_*PA*_ is the ratio of key rate loss in privacy amplification. *Q* represents the gain of the untagged bits, which cannot be measured experimentally, but its upper bounds and lower bounds can be estimated as^34^

2 $$\begin{array}{c}\overline{Q}=\frac{{Q}_{e}}{1-{\rm{\Delta }}-\varepsilon },\\ \underline{Q}=max(0,\frac{{Q}_{e}-{\rm{\Delta }}-\varepsilon }{1-{\rm{\Delta }}-\varepsilon }).\end{array}$$

Then, equation (1) can be rewritten as

3 $$R\ge \frac{1}{L}\{(1-{\rm{\Delta }}-\varepsilon )\underline{Q}-{Q}_{e}fH({e}_{bit})-(1-{\rm{\Delta }}-\varepsilon )\underline{Q}{H}_{PA}\}.$$

If the infinite-intensity decoy states are used, the gain *Q* and the amount of key loss for privacy amplification *QH*_*PA*_ can be given by^34^

4 $$\begin{array}{rcl}Q & = & \sum _{m\mathrm{=(1}-\delta )N}^{m\mathrm{=(1}+\delta )N}\sum _{n\mathrm{=0}}^{\infty }{P}_{in}(m){P}_{n}(m){Y}_{m,n},\\ Q{H}_{PA} & = & \sum _{m\mathrm{=(1}-\delta )N}^{m\mathrm{=(1}+\delta )N}\sum _{n\mathrm{=0}}^{\infty }{P}_{in}(m){P}_{n}(m){Y}_{m,n}H({e}_{ph}^{n}),\end{array}$$

where ${P}_{in}(m)$ is the probability that the input signal of Alice contains *m* photons, and it can be inferred by monitoring the photon distribution at the setup of ID. *P*_*n*_(*m*) is the conditional probability that n photons are emitted by Alice given that m photons enter Alice’s laboratory, which satisfies with a binomial distribution ${P}_{n}(m)={(}_{n}^{m}){\lambda }^{n}{(1-\lambda )}^{m-n}$. Here, *λ* denotes the internal transmittance of Alice’s local laboratory, and it can be set by the IM device. ${e}_{ph}^{n}$ is the phase error rate. *Y*_*m*,*n*_ is the conditional probability that Bob’s detectors click given that m photons enter Alice’s laboratory and n photons are emitted by Alice, and the yield of n-photon state ***Y***_***n***_ is given by

5 $${Y}_{n}=\sum _{m}P\{m|n\}{Y}_{m,n},$$

where $P\{m|n\}$ is the conditional probability that m photons enter Alice’s laboratory given that n photons are emitted by Alice.

According to the analysis in RRDPS-QKD protocol^18^, the phase error rate ${e}_{ph}^{n}$ in equation (4) can be estimated by

6 $${e}_{ph}^{n}\le \frac{n}{L-1},$$

and a threshold photon number ${\nu }_{th} < \frac{L-1}{2}$ is chosen, over which the phase error rate is bounded by 1/2. Then, *QH*_*PA*_ is upper-bounded by

7 $$Q{H}_{PA}\le \sum _{m\mathrm{=(1}-\delta )N}^{m\mathrm{=(1}+\delta )N}\sum _{n\mathrm{=0}}^{{\nu }_{th}}{P}_{in}(m){P}_{n}(m){Y}_{m,n}H({e}_{ph}^{n})+\sum _{m\mathrm{=(1}-\delta )N}^{m\mathrm{=(1}+\delta )N}\sum _{n={\nu }_{th}+1}^{\infty }{P}_{in}(m){P}_{n}(m){Y}_{m,n}H\mathrm{(1/2)}.$$

Thus, the secure key rate of our protocol is estimated by

8 $$\begin{array}{rcl}R & \ge  & \frac{1}{L}\{(1-{\rm{\Delta }}-\varepsilon )Q-{Q}_{e}fH({e}_{bit})-(1-{\rm{\Delta }}-\varepsilon )\\  &  & \,\times (\sum _{m\mathrm{=(1}-\delta )N}^{m\mathrm{=(1}+\delta )N}\sum _{n\mathrm{=0}}^{{\nu }_{th}}{P}_{in}(m){P}_{n}(m){Y}_{m,n}H({e}_{ph}^{n})\\  &  & \,+\sum _{m\mathrm{=(1}-\delta )N}^{m\mathrm{=(1}+\delta )N}\sum _{n={\nu }_{th}+1}^{\infty }{P}_{in}(m){P}_{n}(m){Y}_{m,n}H\mathrm{(1/2)})\}.\end{array}$$

### Numerical simulation

In this section, the performance of our proposed protocol is discussed. For simplicity, we consider the source in the asymptotic case, which means that Alice sends bits to Bob infinitely (i.e. $K\to \infty$), therefore *ε* is close to 0. For any $\delta \in \mathrm{[0,}\,\mathrm{1]}$, the tagged ratio Δ can be calculated by

9 $${\rm{\Delta }}=1-[{\rm{\Phi }}(N+\delta N)-{\rm{\Phi }}(N-\delta N)],$$

where Φ is the cumulative distribution function of the photon for the input pulses. Since it is complicated to calculate numerically, particularly for large *N*, we approximate the photon number distribution by a Gaussian distribution in our numerical simulation. Hence Δ is given as ${\rm{\Delta }}=erfc(\sqrt{\frac{N}{2}}\delta )$, and *δ* is chosen as 0.01 in our simulations^34^.

In the formula of key rate, *Q*_*e*_, *e*_*bit*_ and $\underline{Q}$ play important roles in estimating the final secret key rate R. Here, we use the results in ref.^6^ for *Q*_*e*_ and *e*_*bit*_, they can be measured directly by experiment.

10 $${Q}_{e}=1-(1-{p}_{d}){e}^{-\mu {\eta }_{t}{\eta }_{B}},$$

11 $${e}_{bit}=[{e}_{d}(1-{p}_{d})(1-{e}^{-\mu {\eta }_{t}{\eta }_{B}})+{e}_{0}{p}_{d}]/{Q}_{e},$$

where *p*_*d*_ is the background count rate for the detector, *e*_0_ and *e*_*d*_ are the error probabilities caused by the background and the misalignment, respectively. *μ* is the average intensity of the pulse train. *η*_*B*_ is the efficiency of Bob’s detectors, and *η*_*t*_ is the efficiency of the channel transmission, which is expressed as

12 $${\eta }_{t}={10}^{-\alpha l\mathrm{/10}},$$

where *α* and *l* are the channel transmission loss rate and the transmission distance, respectively. In addition, according to the Poisson limit theorem, heavy attenuation can transform arbitrary photon number distribution into a Poisson-like distribution, so *P*_*n*_(*m*) is approximately written as^37^

13 $${P}_{n}(m)=\frac{{e}^{-\mu }{\mu }^{n}}{n!},$$

when the parameters *N* is larger than 10^6^ and *μ* is less than 1, *μ* = *Nλ*. So in our simulation, the parameters *P*_*n*_(*m*) and *Y*_*m*,*n*_ are approximatively independent of *m*. With the infinite number of decoy states, the yields *Y*_*m*,*n*_ can be accurately estimated as

14 $${Y}_{m,n}=1-(1-{p}_{d}){(1-{\eta }_{t}{\eta }_{B})}^{n}.$$

In our simulations, *λ* is an optimal value by calculations, and the other parameters are listed in Table 1 which are reported in ref.^42^, where *L* represents the pulse train length.

**Table 1:** Simulation parameters for our proposed protocol.

*P*_*d*_	*e*_*d*_	*e*_0_	*η*_*B*_	*α*	*f*
1.7 × 10^−6^ *L*	3.3%	50%	4.5%	0.2 *dB/km*	1.16

**Figure 2:**
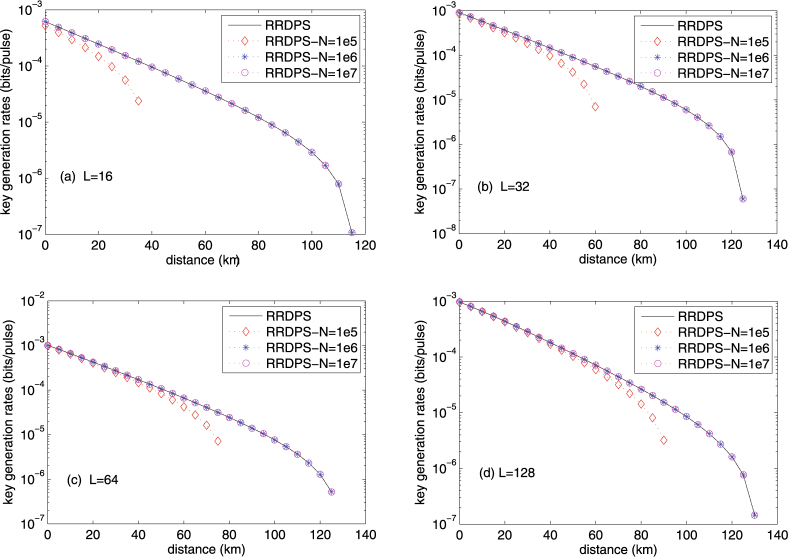
Key rate of the plug-and-play RRDPS-QKD with an untrusted source for *N* = 10^5^, *N* = 10^6^ and *N* = 10^7^. (**a**–**d**) Set the length of the pulse trains as *L* = 16, *L* = 32, *L* = 64 and *L* = 128.

Figure 2 shows the key rates against the transmission distance with different average input photon number N for (a) *L* = 16, (b) *L* = 32, (c) *L* = 64 and (d) *L* = 128. Here, the key rate is a maximized one by optimizing *μ* and *v*_*th*_ for a given transmission distance. From Fig. 2(a–d), we can see that the key rates of the proposed protocol decrease with the increasing transmission distance. The comparison between RRDPS-QKD and the P&P RRDPS-QKD shows that, for the untrusted source, the key rates of the proposed protocol with *N* = 10^6^ and *N* = 10^7^ are similar to that with a trusted source, and is better than the case with *N* = 10^5^. Therefore, *N* is set to 10^6^ in the later simulation analysis.

**Figure 3:**
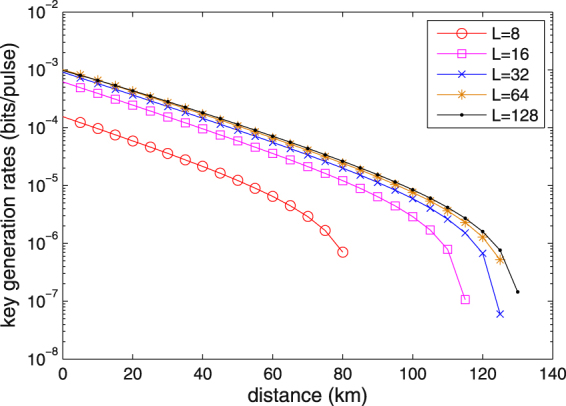
Key rate against the transmission distance for *L* = 16, *L* = 32, *L* = 64 and *L* = 128 with the parameters *δ* = 0.01 and *N* = 10^6^.

Figure 3 shows the key rates of our protocol with *L* = 8, *L* = 16, *L* = 32, *L* = 64 and *L* = 128. The results indicate that the key rates decrease with the increase of distance, and the maximum transmission distance is close to 80 km, 115 km, 125 km, 125 km and 130 km for *L* = 8, *L* = 16, *L* = 32, *L* = 64 and *L* = 128, respectively. Moreover, the maximum key rate and the maximum distance grows with *L*. With a larger *L*, both the key rate and the transmission distance can be improved.

**Figure 4:**
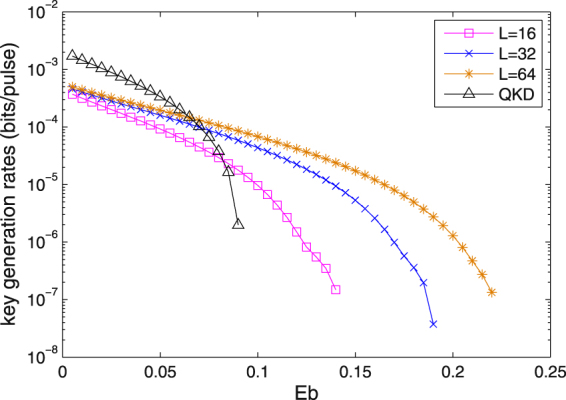
Key rate against the the bit error rate for different *L* with the transmission distance *l* = 30 km.

Figure 4 shows the key rates versus the bit error rate *e*_*bit*_ for P&P RRDPS-QKD protocols with *L* = 16, *L* = 32 and *L* = 64, together with P&P BB84-QKD protocol, where the transmission distance is 30 km. The results show that the key rates of the two protocols decrease as the *e*_*bit*_ increases, and for the P&P RRDPS-QKD protocol, the performance is better when *L* is larger. The comparison results with BB84-QKD show that BB84-QKD has a higher key rate when *e*_*bit*_ is less that 0.065. However, with the increasing *e*_*bit*_, the key rate of BB84-QKD decreases more rapidly, and our protocol has a better key rate performance when *e*_*bit*_ is greater than 0.083. That is, our protocol has a high error tolerance. The P&P RRDPS-QKD protocol keeps the advantage of RRDPS while solving the untrusted source problem.

## Discussion

In this paper, we have proposed the P&P RRDPS-QKD protocol to make RRDPS-QKD protocol be more practical. In the proposed protocol, Bob prepares and sends the strong pulse trains to Alice, then Alice monitors, attenuates, and encodes her bit information on these trains, and sends the attenuated trains back to Bob, who later performs interference measurement to obtain raw keys. After the post processing, Alice and Bob could share the final secret key. We have discussed the security of the protocol and analyzed the tight bound of the key rate with the infinite-intensity decoy states method. With the optimal intensity of the signal states and threshold photon number, we have presented the key generation rates performance. The numerical results show that the proposed protocol can perform as well as the one-way RRDPS-QKD with trusted sources when the intensity of the untrusted source is greater than 10^6^, and the proposed protocol can tolerate more noise than that of P&P BB84-QKD protocol. Moreover, both the key rate and the transmission distance are improved when the pulse number in a train *L* is increased.

## References

[1] Bennett, C. H. & Brassard, G. Quantum cryptography: Public key distribution and coin tossing. In *Proceedings of IEEE International Conference on Computers Systems and Signal Processing*, 175–179 (1984).

[2] Lo H-K, Chau HF (1999). Unconditional security of quantum key distribution over arbitrarily long distances. Science.

[3] Shor PW, Preskill J (2000). Simple proof of security of the BB84 quantum key distribution protocol. Phys. Rev. Lett..

[4] Mayers D (2001). Unconditional security in quantum cryptography. J. Acm.

[5] Lo H-K, Ma X, Chen K (2005). Decoy state quantum key distribution. Phys. Rev. Lett..

[6] Ma XF, Qi B, Zhao Y, Lo HK (2005). Practical decoy state for quantum key distribution. Phys. Rev. A.

[7] Song TT, Qin SJ, Wen QY, Wang YK, Jia HY (2015). Finite-key security analyses on passive decoy-state QKD protocols with different unstable sources. Sci. Rep..

[8] Acin A (2007). Device-independent security of quantum cryptography against collective attacks. Phys. Rev. Lett..

[9] Lo H-K, Curty M, Qi B (2012). Measurement-device-independent quantum key distribution. Phys. Rev. Lett..

[10] Ma X, Razavi M (2012). Alternative schemes for measurement-device-independent quantum key distribution. Phys. Rev. A.

[11] Wang L, Zhao SM, Gong LY, Cheng WW (2015). Free-space measurement-device-independent quantum-key-distribution protocol using decoy states with orbital angular momentum. Chin. Phys. B.

[12] Hwang W-Y, Su H-Y, Bae J (2016). N-dimensional measurement-device-independent quantum key distribution with N + 1 un-characterized sources: zero quantum-bit-error-rate case. Sci. Rep..

[13] Mizutani A, Tamaki K, Ikuta R, Yamamoto T, Imoto N (2014). Measurement-device-independent quantum key distribution for Scarani-Acin-Ribordy-Gisin 04 protocol. Sci. Rep..

[14] Mao QP, Zhao SM, Wang L, Qian CC, Chen HW (2017). Wavelength division multiplexing for measurement-device-independent quantum key distribution. Chin. J. Quantum Electron..

[15] Gottesman D, Lo HK, Lutkenhaus N, Preskill J (2004). Security of quantum key distribution with imperfect devices. Quantum Inf. Comput..

[16] Yin H-L, Fu Y, Mao Y, Chen Z-B (2016). Security of quantum key distribution with multiphoton components. Sci. Rep..

[17] Kato G, Tamaki K (2016). Security of six-state quantum key distribution protocol with threshold detectors. Sci. Rep..

[18] Sasaki T, Yamamoto Y, Koashi M (2014). Practical quantum key distribution protocol without monitoring signal disturbance. Nature.

[19] Mizutani A, Imoto N, Tamaki K (2015). Robustness of the round-robin differential-phase-shift quantum-key-distribution protocol against source flaws. Phys. Rev. A.

[20] Cao Z, Yin ZQ, Han ZF (2016). Trustworthiness of measurement devices in round-robin differential-phase-shift quantum key distribution. Phys. Rev. A.

[21] Yin H-L, Fu Y, Mao Y, Chen Z-B (2016). Detector-decoy quantum key distribution without monitoring signal disturbance. Phys. Rev. A.

[22] Zhang YY (2016). Practical round-robin differential phase-shift quantum key distribution. Opt. Express.

[23] Liu L, Guo F-Z, Qin S-J, Wen Q-Y (2017). Round-robin differential-phase-shift quantum key distribution with a passive decoy state method. Sci. Rep..

[24] Wang L, Zhao S (2017). Round-robin differential-phase-shift quantum key distribution with heralded pair-coherent sources. *Quantum Inf*. Processing.

[25] Zhang Z, Yuan X, Cao Z, Ma X (2017). Practical round-robin differential-phase-shift quantum key distribution. New J. Phys..

[26] Hu K, Mao QP, Zhao SM (2017). Round robin differential phase shift quantum key distribution using heralded single photon source and detector decoy. Acta Opt. Sin..

[27] Guan JY (2015). Experimental passive round-robin differential phase-shift quantum key distribution. Phys. Rev. Lett..

[28] Takesue H, Sasaki T, Tamaki K, Koashi M (2015). Experimental quantum key distribution without monitoring signal disturbance. Nature Photon..

[29] Wang S (2015). Experimental demonstration of a quantum key distribution without signal disturbance monitoring. Nature Photon..

[30] Li Y-H (2016). Experimental round-robin differential phase-shift quantum key distribution. Phys. Rev. A.

[31] Wang X-B, Hiroshima T, Tomita A, Hayashi M (2007). Quantum information with Gaussian states. Phys. Rep..

[32] Muller A (1997). “Plug and play” systems for quantum cryptography. Appl. Phys. Lett..

[33] Stucki D, Gisin N, Guinnard O, Ribordy G, Zbinden H (2002). Quantum key distribution over 67 km with a plug& play system. New J. Phys..

[34] Zhao Y, Qi B, Lo HK (2008). Quantum key distribution with an unknown and untrusted source. Phys. Rev. A.

[35] Peng X, Xu BJ, Guo H (2010). Passive-scheme analysis for solving the untrusted source problem in quantum key distribution. Phys. Rev. A.

[36] Zhao Y, Qi B, Lo HK, Qian L (2010). Security analysis of an untrusted source for quantum key distribution: passive approach. New J. Phys..

[37] Xu FH (2015). Measurement-device-independent quantum communication with an untrusted source. Phys. Rev. A.

[38] Gisin N, Fasel S, Kraus B, Zbinden H, Ribordy G (2006). Trojan-horse attacks on quantum-key-distribution systems. Phys. Rev. A.

[39] Lo H-K, Preskill J (2007). Security of quantum key distribution using weak coherent states with nonrandom phases. Quantum Inf. Comput..

[40] Cao Z, Zhang Z, Lo H-K, Ma X (2015). Discrete-phase-randomized coherent state source and its application in quantum key distribution. New J. Phys..

[41] Pawlowski M (2009). Information causality as a physical principle. Nature.

[42] Gobby C, Yuan ZL, Shields AJ (2004). Quantum key distribution over 122 km of standard telecom fiber. Applied Physics Letters.

